# Comprehensive Analysis of Glycolysis-Related Genes for Prognosis, Immune Features, and Candidate Drug Development in Colon Cancer

**DOI:** 10.3389/fcell.2021.684322

**Published:** 2021-08-06

**Authors:** Zhongqi Cui, Guifeng Sun, Ramesh Bhandari, Jiayi Lu, Mengmei Zhang, Rajeev Bhandari, Fenyong Sun, Zhongchen Liu, Shasha Zhao

**Affiliations:** ^1^Department of Clinical Laboratory, Shanghai Tenth People’s Hospital, Tongji University, Shanghai, China; ^2^Department of Pathology, Universal College of Medical Sciences, Bhairahawa, Nepal; ^3^Department of General Surgery, Universal College of Medical Sciences, Bhairahawa, Nepal; ^4^Department of Gastrointestinal Surgery, Shanghai Tenth People’s Hospital Affiliated to Tongji University, Shanghai, China

**Keywords:** glycolysis, colon cancer, prognosis model, immune microenvironment, tumor mutation burden, chemotherapy response

## Abstract

The dysregulated expression of glycolysis-related genes (GRGs) is closely related to the occurrence of diverse tumors and regarded as a novel target of tumor therapy. However, the role of GRGs in colon cancer is unclear. We obtained 226 differential GRGs (DE-GRGs) from The Cancer Genome Atlas (TCGA) database. Cox regression analysis was used to construct a DE-GRG prognostic model, including P4HA1, PMM2, PGM2, PPARGC1A, PPP2CB, STC2, ENO3, and CHPF2. The model could accurately predict the overall survival rate of TCGA and GSE17536 patient cohorts. The risk score of the model was closely related to a variety of clinical traits and was an independent risk factor for prognosis. Enrichment analysis revealed the activation of a variety of glycolysis metabolism and immune-related signaling pathways in the high-risk group. High-risk patients displayed low expression of CD4+ memory resting T cells and resting dendritic cells and high expression of macrophages M0 compared with the expression levels in the low-risk patients. Furthermore, patients in the high-risk group had a higher tumor mutation load and tumor stem cell index and were less sensitive to a variety of chemotherapeutic drugs. Quantitative reverse transcription polymerase chain reaction and immunohistochemistry analyses validated the expression of eight GRGs in 43 paired clinical samples. This is the first multi-omics study on the GRGs of colon cancer. The establishment of the risk model may benefit the prognosis and drug treatment of patients.

## Introduction

Colon cancer is the most prevalent gastrointestinal malignancy. It is the third-ranked malignancy-related death globally (Arnold et al., 2020). According to the National Cancer Institute and GLOBOCAN data, over one million new cases of colorectal cancer (CRC) are diagnosed annually, and the estimated death rate is 33%. Colon cancer is a multifactorial disease, and its causes could include genetic predisposition, lifestyle changes, and diet (Pilleron et al., 2019; Vuik et al., 2019). Despite recent advancements in medicine, diagnostics tools, and surgical techniques, the annual incidence and mortality rates of colon cancer continue to increase appreciably, and the 5-year survival rate is less than 15% (Kalyan et al., 2018; Ladabaum et al., 2020). Therefore, it is crucial to discover new biomarkers and therapeutic drug targets for the rapid diagnosis and treatment of colon cancer.

Increasing evidence on cellular metabolism has revealed the alteration in energy metabolism from oxidative phosphorylation (OXPHOS) to aerobic glycolysis that occurs in response to the increased bioenergetic demands of rapidly proliferating and invading cancerous cells. This altered cellular energy metabolism is an important hallmark for cancer development (Cairns et al., 2011; Hanahan and Weinberg, 2011). Unlike normal cells, tumor cells also derive the energy from glucose glycolysis, which is termed as the Warburg effect (Gatenby and Gillies, 2004; Vander Heiden et al., 2009). Lactate produced by the Warburg effect creates a suitable acidic tumor microenvironment (TME) that facilities tumor progression, invasion, and immune response (Corbet and Feron, 2017). Therefore, targeting the glycolysis pathway could be beneficial for tumor prognosis and effective cancer treatment (DeBerardinis et al., 2008; Martinez-Outschoorn et al., 2017).

Glycolysis can alter the biological function of immune cells within the TME and can help cancer cells escape the host body defense mechanism, facilitating cancer progression (Gill et al., 2016). However, only a few comprehensive analyses have explored the role and relationship of tumor glycolysis and immune cell infiltration in colon cancer development.

In the current study, we systematically analyzed the glycolysis-related genomic expression profile in colon adenocarcinoma (COAD) samples from The Cancer Genome Atlas (TCGA) database. The 226 differential glycolysis-related genes (DE-GRGs) that were identified included 17 survival-related GRGs. A risk model incorporating eight GRGs (P4HA1, STC2, PPARGC1A, PPP2CB, ENO3, CHPF2, PMM2, and PGM2) was constructed. The model displayed a high predictive value for the overall survival (OS) rate of colon cancer in both the training and validation groups. Furthermore, the risk model was closely related to a variety of clinical risk factors and could be used as an independent prognostic index for patients with colon cancer. This model may also reflect the dysregulation of immune cell infiltration, the change of gene copy number, and the increase of tumor mutation load and stem cell index. The findings indicate the utility of the model as a powerful tool for the prognosis and treatment of colon cancer.

## Materials and Methods

### Data Collection and Acquisition of Glycolysis-Related Genes

The expressed gene RNA-sequencing dataset (fragments per kilobase of transcript per million [FPKM] value) and corresponding TCGA clinical information of 473 COAD and 71 non-tumor tissue samples were extracted^1^. A total of 298 GRGs, of which 226 were DE-GRGs, were identified by gene set enrichment analysis (GSEA) of glycolysis-related pathways (Supplementary Figure 1).

### Identification of Differential Glycolysis-Related Gene and Function Analyses

Differential glycolysis-related genes in TCGA COAD tumor tissues and adjacent non-tumor tissues with a false discovery rate (FDR) < 0.05 were identified using the limma R package. The 226 DE-GRGs identified were displayed by Venn diagram (Supplementary Table 4). Biological process (BP) and the Kyoto Encyclopedia of Genes and Genomes (KEGG) pathway analyses were performed by the clusterProfiler R package (Liao et al., 2019) to identify the functionally enriched genes and classify the gene clusters. A *q*-value < 0.05 was considered statistically significant.

### Construction and Visualization of Protein–Protein Interaction Network Module

The PPI of the 226 DE-GRGs was analyzed in the String online database^2^ as previously described (Szklarczyk et al., 2019). The network was visualized by Cytoscape 3.7.2 software^3^. The key modules in the PPI network were filtered according to the score and the criterion of node counts >5 (Bader and Hogue, 2003) using the MCODE plug-in in Cytoscape. A *p*-value ≤ 0.05 denoted a significant difference.

### Construction of Prognostic Model

Key GRGs identified using the survival R package were further subjected to univariate Cox regression analysis to screen and identify significant prognosis candidates. The candidate genes were incorporated into the multivariate Cox risk model. Eight GRGs associated with significant prognosis potential were retained in the process of multiple calculations. The risk score for each patient was calculated as follows:

Riskscore=∑regressioncoefficient(genei)×expressionvalue(genei)

Colon adenocarcinoma patients in the training group were categorized into the low- and high-risk groups based on their median risk score. The OS rates of the two subgroups were compared by the Kaplan–Meier method and the log-rank test. A receiver operating characteristic (ROC) curve was constructed by SurvivalROC R package to evaluate the prognosis ability of the aforementioned model. In addition, 177 COAD patient samples from the GSE17536 dataset (Li et al., 2018) were used to validate the predictive ability of this prognostic model. Finally, the RMS-R package was used to construct a nomogram with calibration plots to show the likelihood OS rate.

### Patients and Tissue Sampling

A total of 43 pairs of colon tumor tissues and adjacent non-tumor tissues were collected from patients who underwent colon cancer surgery at Shanghai Tenth People’s Hospital from September 2019 to June 2020. After surgical resection, the colon cancer tissue samples were immediately cleaned in normal saline and cryopreserved. Prior to the surgery, none of the patients had received radiation and chemotherapy. This study was sanctioned by the Institutional Research Ethical Committee (IREC) of Shanghai Tenth People’s Hospital of Tongji University of Medicine, Shanghai. All patients provided their verbal and written consent.

### RNA Extraction and Quantitative Reverse Transcription Polymerase Chain Reaction Analysis

Total RNA from the colon cancer tissue and CRC cell lines was extracted using TRIzol reagent (Invitrogen, Thermo Scientific, Shanghai, China). Total mRNA was reverse-transcribed into cDNA using the prime script Reverse Transcription reagent kit (TaKaRa Bio, Shiga, Japan). Relative expression of eight potentially prognostic GRG markers was determined by qRT-PCR using the SYBR Green reagent kit (TaKaRa Bio) in an ABI 7500 PCR system (Applied Biosystems). Glyceraldehyde 3-phosphate dehydrogenase (GAPDH) was used to normalize gene expression. Primer sequences are listed in Supplementary Table 1.

### Immunohistochemistry Staining of Colon Cancer Tissue Sections

Paraffin-embedded colon cancer tissue was cut into 5-μm-thin sections. The sections were deparaffinized and rehydrated. Antigen retrieval was performed by exposure of the sections to 3% hydrogen peroxide along with normal goat serum. Antigen retrieval tissue sections were initially treated with antibody to stanniocalcin-2 (STC2, 1:200 dilution), prolyl 4-hydroxylase subunit alpha 1 (P4HA1, 1:200), protein phosphatase 2 catalytic subunit beta (PPP2CB, 1:200 or 1:250), peroxisome proliferator-activated receptor gamma coactivator 1-alpha (PPARGC1A, 1:250), enolase 3 (ENO3, 1:200), chondroitin polymerizing factor 2 (CHPF2, 1:500), phosphoglucomutase 2 (PGM2, 1:200), and phosphomannomutase 2 (PMM2, 1:200) (all from Abcam, Cambridge, United Kingdom). The sections were subsequently treated with biotinylated secondary rabbit antibodies conjugated with streptavidin–horseradish peroxidase (1:200). The binding of secondary antibody was revealed by reaction with added 3,3’-diaminobenzidine (DAB) followed by counterstaining with hematoxylin. The stained slides were visualized and photographed by optical microscopy (Leica, Tokyo, Japan). Image-Pro Plus 6.0 Software (Media Cybernetics, Rockville, MD, United States) was used to analyze protein expression in the stained tissue sections.

### Cell Culture and Transient Transfection

HCT116 and SW480 human CRC cell lines were cultured at 37°C in a 5% CO_2_ incubator in Roswell Park Memorial Institute (RPMI) 1,640 medium or Dulbecco’s modified Eagle’s medium (both from Gibco, Grand Island, NY, United States) supplemented with 10% fetal bovine serum (FBS). For transient transfection, prior to the day of transfection 2.5 × 10^5^/ml of HTC116 and SW480 cells were seeded in six-well plates and incubated overnight to allow development of 40 to 60% confluent growth. These cells were transfected with small interfering RNA (siRNA) to STC2 (Gene Pharma, Shanghai, China) using Lipofectamine 2000 (Invitrogen, Carlsbad, CA, United States) and incubated further for 48 h. Target sequences for siRNAs were as follows:

*STC2-si1: GCGUGUUUGAAUGUUUCGATT* and *STC2-si2: GGGUGAUAGUGGAGAUGAUTT*.

### Cell Proliferation and Colony Formation Assay

Proliferation of STC2-siRNA-treated HCT116 and SW480 cells was determined using the Cell Counting Kit-8 (Dojindo Molecular Technologies, Inc., Rockville, MD, United States) as previously described (Liu et al., 2019). A clonogenic assay was performed to measure and the monitor the colony-forming capabilities of STC2-siRNA CRC cell (Zhen et al., 2019).

### Cell Invasion and Migration Assay

Matrigel precoated Transwell inserts were placed into 24-well plate filled with 500 μl of culture medium supplemented with 10% FBS. Aliquots (400 μl containing 5 × 104 SW480 or HCT116 cells) of STC2-siRNA-treated cell suspensions were added to the upper chamber of Transwell units and incubated at 37°C for 20–24 h in a 5% CO_2_ incubator. Cells that traversed the membrane separating the upper and lower chambers of each unit (i.e., invading cells) were fixed with 4% paraformaldehyde (PFA) for 15 min at room temperature and stained with 0.1% crystal violet. Cells that did not invade were gently removed from the membrane surface exposed to the upper chamber using a wet cotton swab. Each Transwell unit was allowed to dry and examined by inverted microscopy (Leica). Quantitative analysis of invaded cell was performed using ImageJ software (NIH, Bethesda, MD, United States).

For the cell migration assay, SW480 and HCT116 cells were transfected with STC2-siRNA and cultured on solid medium. Twenty-four hours later, a wound was created horizontally by gently pressing a 200-μl sterile micropipette tip on the surface of the confluent cell monolayer on six-well plates. Each well was washed twice with serum-free medium. Photographs were taken with an inverted microscope (Leica) at 0 and 24 h. The cell migration rate was determined by time-lapse analysis using ImageJ software.

### Evaluation of Immune Cell Infiltration

The CIBERSORT^4^ online bioinformatics analytical tool was used to estimate and distinguish 22 commonly infiltrating immune cells that included B cells, B memory cells, plasma cells, CD8+ and naïve CD4+ T cells, natural killer cells, macrophages, dendritic cells, and mast cells in COAD patients with low- and high-risk scores (Newman et al., 2015).

### Estimation of Tumor Mutation Burden, Copy Number Variation, and Tumor Stemness

Tumor mutation burden (TMB) on the target gene was calculated by dividing the total number of mutations by the size of the coding region of the target gene. The GenVisR R package was used for the evaluation and analysis of the top 30 frequently mutated genes among the low- and high-risk groups (Skidmore et al., 2016). The copy number variation (CNV) in these groups of patients was calculated using chi-square test (Feng et al., 2020). The Rcircos R package was used to construct Circos plots to determine the CNV in chromosomes (Zhang et al., 2013). Utilization of the mRNA expression-based stemness index (mRNAsi) and epidermal growth factor receptor (EGFR)-mRNAsi data obtained from Tathiane Malta, University of São Paulo, was used to estimate the tumor stemness index to assess the dedifferentiation potential of oncogenic cells (Malta et al., 2018).

### Prediction of Responses of Antineoplastic Drugs and Small Candidate Molecules

Based on the Genomics of Drug Sensitivity in Cancer (GDSC)^5^, the efficacy of distinct antineoplastic drugs on COAD TCGA samples was determined using the pRRophetic R package. The half-maximal inhibitory concentration (IC50) of a specific chemotherapeutic drug was obtained by ridge regression analysis. The prediction accuracy was measured by the 10-fold cross-validation of the GDSC cell line expression profile data. In addition, the Broad Institute Connectivity Map (CMap^6^) was used to predict the small candidate molecules based on the top 1,000 differentially expressed genes (Subramanian et al., 2017).

### Statistical Analyses

All statistical analyses were performed using R software (version 3.6.1). The Wilcoxon test was used for the comparison of two independent non-parametric samples. The Kruskal–Wallis test was used for multiple independent samples. The Kaplan–Meier survival curves were compared with the log-rank test. Univariate and multivariate Cox proportional hazard regressions were performed to identify independent prognostic factors associated with OS. A *p*-value < 0.05 was considered statistically significant.

## Results

**FIGURE 1:**
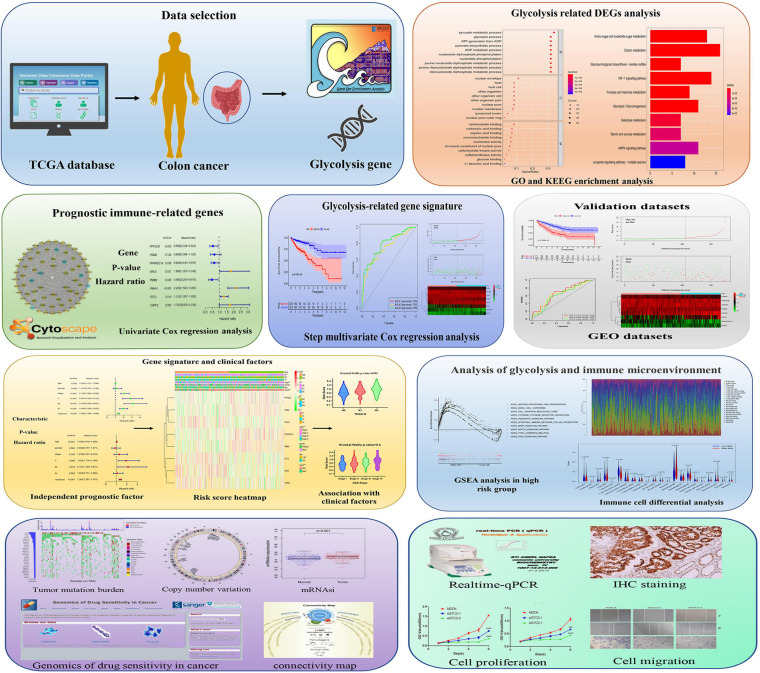
Schematic diagram of the comprehensive analysis of glycolysis-related genes (GRGs) for prognosis prediction and drug selection in colon adenocarcinoma (COAD) patients.

As increased aerobic glycolysis activity plays a significant role in cancer development, we explored the prognostic importance of GRGs in colon cancer (Figure 1). TCGA data of a total of 473 COAD patients and 177 patients from the Gene Expression Omnibus (GEO) (GSE17536) cohort were included. The demographics and clinical details of the patients are summarized in Table 1.

**TABLE 1:** Clinical characteristics of the colon cancer patients used in this study.

	TCGA cohort	GSE17536
**Number of patients**	473	177
**Age in years (median, range)**	69 (31–90)	66 (26–92)
**Gender (%)**		
Female	215 (45.5%)	81 (45.8%)
Male	258 (54.5%)	96 (54.2%)
**Grade (%)**		
1	NA	16 (9.1%)
2	NA	133 (75.7%)
3	NA	27 (15.3%)
4	NA	0 (0%)
**Stage (%)**		
I	76 (16.1%)	24 (13.6%)
II	177 (37.4%)	57 (32.2%)
III	125 (26.4%)	57 (32.2%)
IV	62 (13.1%)	39 (22.0%)
T		
I	10 (2.11%)	NA
II	77 (16.3%)	NA
III	307 (64.9%)	NA
IV	56 (9.72%)	NA
M		
M0	333 (70.4%)	NA
M1	62 (13.1%)	NA
MX	49 (10.4%)	NA
N		
N0	268 (56.7%)	NA
N1	103 (21.8%)	NA
N2	80 (16.9%)	NA

*NA, not applicable; TCGA, The Cancer Genome Atlas.*

### Identification of Differential Glycolysis-Related Genes and Functional Annotations

**FIGURE 2:**
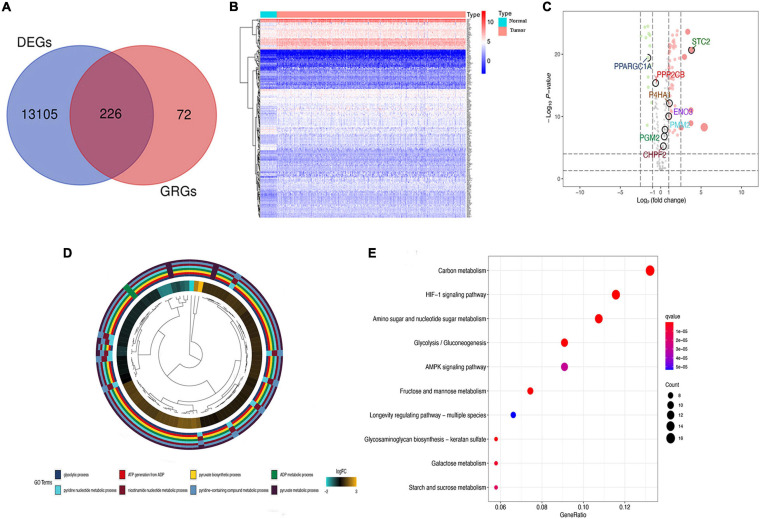
Differential glycolysis-related genes (DE-GRGs) in The Cancer Genome Atlas (TCGA) patients. **(A)** Venn diagram of DE-GRGs among tumor and non-tumor TCGA samples. **(B)** Heatmap of expression of DE-GRGs in colon adenocarcinoma (COAD) sample. **(C)** Volcano plot of DE-GRG expression in tumors and normal tissue samples. Green and red dots indicate downregulated and upregulated GRGs, respectively. **(D)** Biological process (BP) analysis of DE-GRGs. **(E)** Kyoto Encyclopedia of Genes and Genomes (KEGG) pathway enrichment analysis of DE-GRGs.

The primary screening and analysis of TCGA samples by GSEA identified 226 DE-GRGs in COAD samples compared with normal tissues (Figure 2A). Of these, 154 were overexpressed and 72 were underexpressed (Figures 2B,C; Supplementary Table 2). The DE-GRGs were significantly augmented in BP terms related to carbohydrate metabolism, nucleotide metabolism, AMP-activated protein kinase, and hypoxia-inducible factor signaling pathway (Figure 2D and Supplementary Table 2). KEGG pathway analysis shows that DE-GRGs are involved in HIF-1 signaling pathway, glycolysis/gluconeogenesis, AMPK signaling pathway, and galactose metabolism (Figure 2E and Supplementary Table 3).

### Establishment of Protein–Protein Interaction Network and Selection of Prognosis-Related Glycolysis-Related Genes

**FIGURE 3:**
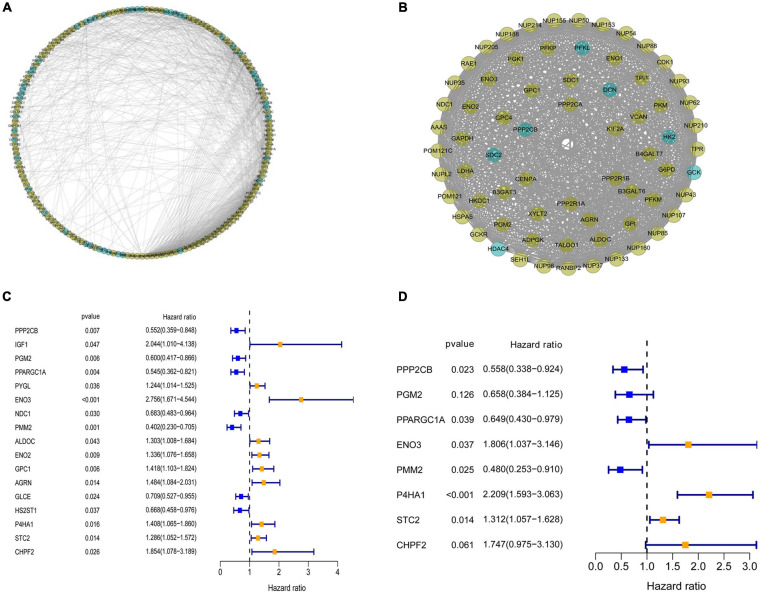
Protein–protein interaction (PPI) network construction and selection of prognosis–related glycolysis-related genes (GRGs). **(A)** PPI network of differential GRGs (DE-GRGs). **(B)** Critical module constructed from PPI network. Green circles indicate downregulation with more than two-fold change. Yellow circles indicate upregulation with more than two-fold change. **(C)** Univariate Cox regression analysis with preliminary identified prognostic related GRGs. **(D)** Multivariate Cox regression analysis with prognosis-related GRGs.

To investigate the role of GRGs in COAD, a PPI network with 177 nodes and 1,216 edges was constructed using Cytoscape String software (Figure 3A). In addition, a co-expression network with 69 nodes and 701 edges was created using the MCODE tool (Figure 3B) to identify the key modules of GRGs. To explore the prognostic value of these GRGs, univariate Cox regression analysis was performed. Seventeen prognostic-associated candidate hub GRGs were revealed (Figure 3C). These candidate hub GRGs were analyzed by multiple stepwise Cox regression to investigate their impact on patient survival and clinical outcomes. Eight hub GRGs were independent predictors in COAD patients (Figure 3D and Table 2).

**TABLE 2:** Glycolysis related genes Multivariate Cox regression results.

Multivariate cox analysis		
GRGs	HR	*P* value
PPP2CB	0.558(0.338–0.924)	0.023
PGM2	0.658(0.384–1.125)	0.126
PPARGC1A	0.649(0–430-0.979)	0.039
EN03	1.806(1.037–3.146)	0.037
PMM2	0.480(0.253–0.910)	0.025
P4HA1	2.209(1.593–3.063)	<0.001
STC2	1.312(1.057–1.628)	0.014
CHPF2	1.747(0.975–3.130)	0.061

### Prognosis-Related Glycolysis-Related Genes Risk Score Model Construction and Validation

A prediction model incorporating a prognosis-related signature of the eight hub GRGs was determined with the following calculation of the risk score:

Riskscore=(-0.5826*ExpPPP2CB)+(-0.4192*ExpPGM2)

+(-0.4322*ExpPPARGC1A)+(0.5911*ExpENO3)+

(-0.735*ExpPMM2)+(0.7924*ExpP4HA1)+

(0.2715*ExpSTC2)+(0.5578*ExpCHPF2)

**FIGURE 4:**
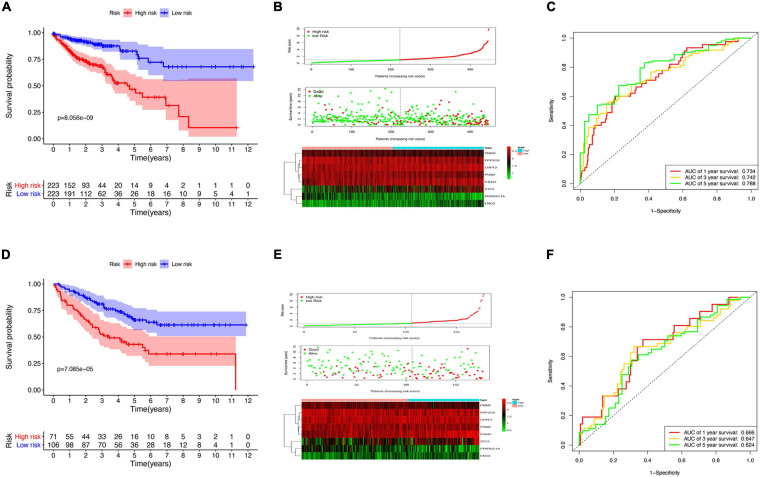
Risk model construction and validation. **(A)** Kaplan–Meier plots illustrating the worse overall survival (OS) rate in the high-risk group compared with the low-risk group patients in The Cancer Genome Atlas (TCGA) cohort. **(B)** Expression heatmap, risk score distribution, and survival status in TCGA cohort. **(C)** Time-dependent area under the curve (AUC) of the receiver operating characteristic (ROC) curve of the 1-, 3-, and 5-year OS rates of TCGA patients. **(D)** Kaplan–Meier plots of worse OS in the high-risk patients compared with low-risk patients in the GSE17536 cohort. **(E)** Expression heatmap, risk score distribution, and survival status in the GSE17536 cohort. **(F)** Time-dependent AUC ROC curve of 1-, 3-, and 5-year OS rates of patients in the GSE17536 cohort.

Survival analysis in the low- and high-risk groups was determined using the Kaplan–Meier survival plot. The analysis revealed a relatively low OS rate in patients with high-risk score compared with the rate in the low-risk group of patients (Figure 4A). The risk scores, survival scores, and heatmap of GRG expression among the low- and high-risk patients are presented in Figure 4B. In addition, the area under the curve (AUC) analysis of COAD patients at 1, 3, and 5 years revealed respective OS rates of 73.4, 74.2, and 78.8% (Figure 4C). To verify the effectiveness of this model, a risk score was calculated in the GSE17536 cohort of patients, and they were classified into the high-risk group (*n* = 71) and low-risk group (*n* = 106) based on median risk score. Surprisingly, the results obtained from the GSE17536 cohort revealed that the high-risk score patients had lower OS rates than the patients with low-risk score, which was identical to the results obtained in TCGA cohort analysis (Figures 4D–F).

### The Glycolysis-Related Gene Signature Confers Additional Prognostic Power for Colon Adenocarcinoma Patients

**FIGURE 5:**
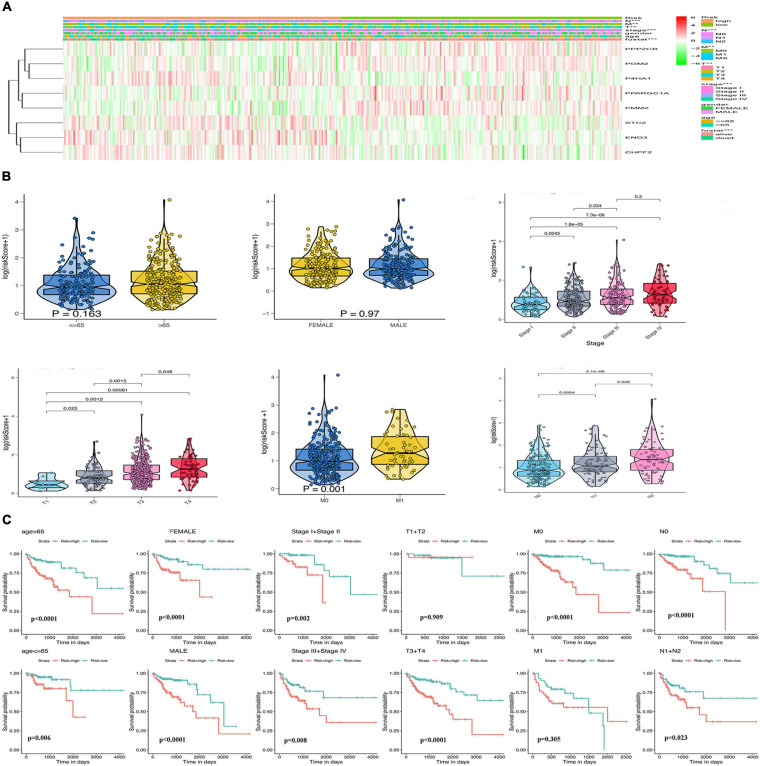
Relationship of the GRG signature with clinicopathological characteristics in The Cancer Genome Atlas (TCGA) data. **(A)** Heatmap of the differential expression of the eight GRGs. **(B)** Box plots showing risk score distribution of different clinical characteristics of the colon adenocarcinoma (COAD) tumors. **(C)** The GRG risk model predicts the overall survival (OS) rate of patients with colon cancer in multiple clinical subgroups.

Considering the importance of glycolysis in the prognosis of colon cancer, we further analyzed the relationship between the eight GRGs and the clinical characteristics of colon cancer, including age, sex, tumor stage, and TMN stage. The heatmap (Figure 5A) indicated that the expression of CHPF2, ENO3, STC2, and P4HA1 was upregulated in the high-risk group, whereas that of PPP2CB, PGM2, PMM2, and PPARGC1A was downregulated in the high-risk group. Furthermore, a substantial difference in risk scores was evident for the different T, N, and M grades and tumor stages. The risk score increased with tumor progression (Figure 5B and Supplementary Figure 2A). To better predict the prognosis of the model, stratification analysis was used to confirm whether the model retained the ability to predict OS in different clinical subgroups. The OS rate of colon cancer patients with high-risk score, compared with patients in the low-risk group, was worse in the T3 + T4 and M0 groups (Figure 5C). Similarly, the risk model was good at predicting the OS rate of patients >65 or ≤65 years of age, male or female patients, stage I + II or III + IV, grade 1 or grade 2 + 3, and N0 or N1 (Figure 5C and Supplementary Figure 2B). The collective data indicate that the model may be a good prognostic index for patients with COAD.

### Gene Set Enrichment Analysis Enrichment and Immune Cell Infiltration Analysis in the Cancer Genome Atlas and Gene Expression Omnibus Datasets

**FIGURE 6:**
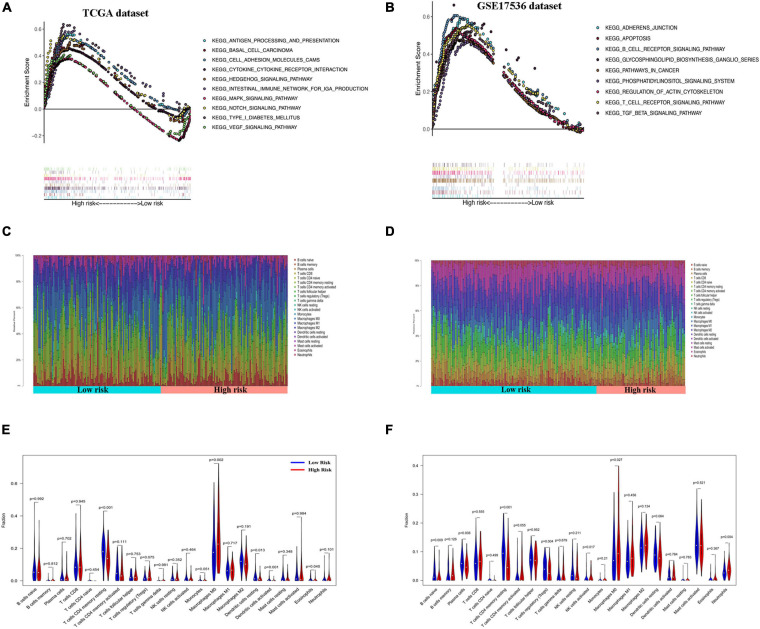
Gene set enrichment analysis (GSEA) and immune cell infiltration analyses in low- and high-risk patients. **(A,B)** Kyoto Encyclopedia of Genes and Genomes (KEGG) enrichment of low- and high-risk groups by GSEA in The Cancer Genome Atlas (TCGA) cohort **(A)** and GSE17536 cohort **(B)**. Only gene sets with false discovery rate (FDR) *q* < 0.05 were considered significant. **(C,D)** Relative proportion of infiltrating immune cells in low- and high-risk colon adenocarcinoma (COAD) patients in TCGA cohort **(C)** and GSE17536 cohort **(D)**. **(E,F)** Comparative demonstration of the distinct infiltrating immune cells in low- and high-risk COAD patients in TCGA cohort **(E)** and GSE17536 cohort **(F)**.

Gene set enrichment analysis of the GEO and TCGA databases revealed the aberrant activation of multiple signaling pathways. These included oxidative phosphorylation, chemokine receptor interaction, glycerol metabolism, chemokine signal pathway, metabolism or exogenous substances through cytochrome P450, and transforming growth factor-beta signaling pathways in high-risk COAD patients (Figures 6A,B).

CIBERSORT along with the LM22 matrix was used to assess immune cell infiltration in the low- and high-risk groups of COAD patients. The GEO and TCGA and dataset analyses indicated that CD4+ and CD8+ T cells, macrophages, and mast cells were the predominant infiltrating immune cells in COAD (Figures 6C,D). Furthermore, infiltrations of resting CD4+ memory T cells, resting dendritic cells, and activated dendritic cells were significantly downregulated in the high-risk patients. In contrast, the distributions of M0 macrophages and eosinophils were opposite in the low- and high-risk TCGA groups (Figure 6E). Similarly, in the GSE17536 dataset, the infiltrating immune cells comprised significantly low proportions of CD4+ memory resting T cells and resting dendritic cells and high infiltration of M0 macrophages compared with those in the low-risk group (Figure 6F). However, there was no significant difference in the infiltrations of activated dendritic cells and eosinophils between the low- and high-risk groups.

### Estimation of Mutational Load, Genetic Variation, Stemness Index, and Sensitivity to Antineoplastic Drugs

**FIGURE 7:**
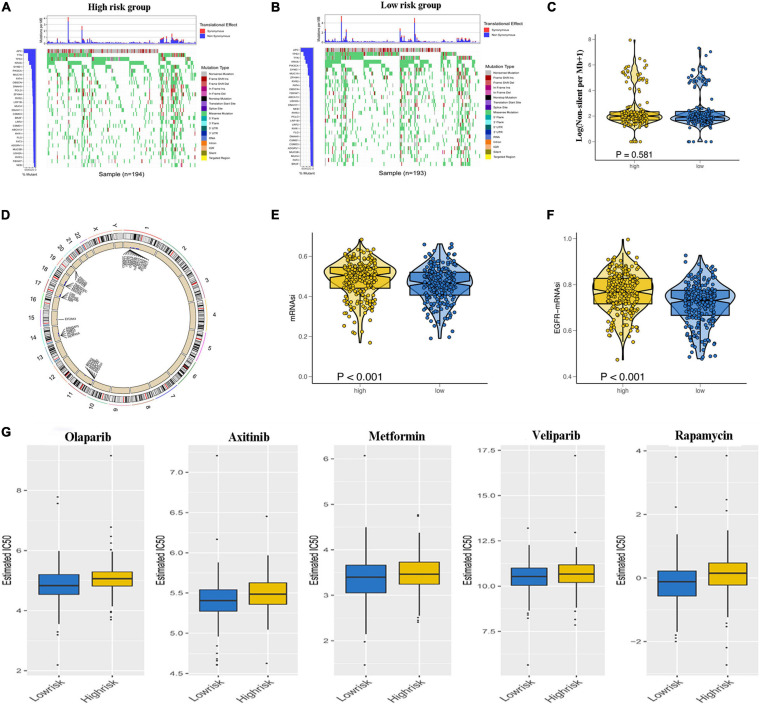
Relationship of glycolysis-related gene (GRG) risk group with tumor mutation burden, copy number variation, tumor stemness, and chemotherapy response. **(A,B)** Waterfall plots demonstrating the higher frequency of mutated genes in the high-risk and low-risk groups. **(C)** Difference in total tumor mutation burden in low- and high-risk colon adenocarcinoma (COAD) patients. **(D)** Distinct genes with different copy number variations due to gains or loss of nucleotides mainly on chromosomes 1, 11, 14, 17, 18, and 19 **(E,F)**. Difference in tumor stemness by the mRNAsi **(E)** and EGFR mRNAsi **(F)**. **(G)** Estimated IC50 indicating the efficiency of chemotherapy to GRGs in low- and high-risk patients of olaparib, veliparib, axitinib, metformin, and rapamycin.

To explore the underlying reasons for the difference in prognosis of patients in the low- and high-risk groups, we analyzed the TMB, CNV, and tumor stemness among the low- and high-risk groups. Waterfall plots were constructed to demonstrate the TMB and to differentiate TMB among the two risk groups. As shown in Figures 7A,B, most genes displayed higher mutation frequencies in the high-risk group. These genes included TP53, TTN, and KRAS. We calculated the TMB for each patient in both risk groups. The mean TMB was not significantly different between the two groups (*p* > 0.05; Figure 7C). In addition, distinct variations in CNV were evident in 920 genes predominantly located in chromosomes 1, 11, 14, 17, 18, and 19 (Figure 7D). A one-class logistic regression analysis was performed to calculate (mRNAsi) and epigenetic regulation-based stemness index (EREG-mRNAsi). The high-risk group had higher stemness indices compared with the low-risk group (Figures 7E,F). To evaluate the therapeutic effect of the risk model, the pRRophetic R package was used to analyze data downloaded from the GDSC database for the prediction of the sensitivity of distinct chemotherapeutic drugs on GRGs. Estimated IC50 values indicated that the low-risk patients were more sensitive to the anticancer drugs olaparib, veliparib, axitinib, metformin, and rapamycin (*p* < 0.05; Figure 7G).

### Glycolysis-Related Gene Prognostic Model Is an Independent Prognostic Factor for Colon Adenocarcinoma Patients

To further judge whether the GRG model can be used as an independent factor to predict the prognosis of patients with colon cancer, Cox regression analysis was performed to reveal an association between risk score and OS of the patients.

**FIGURE 8:**
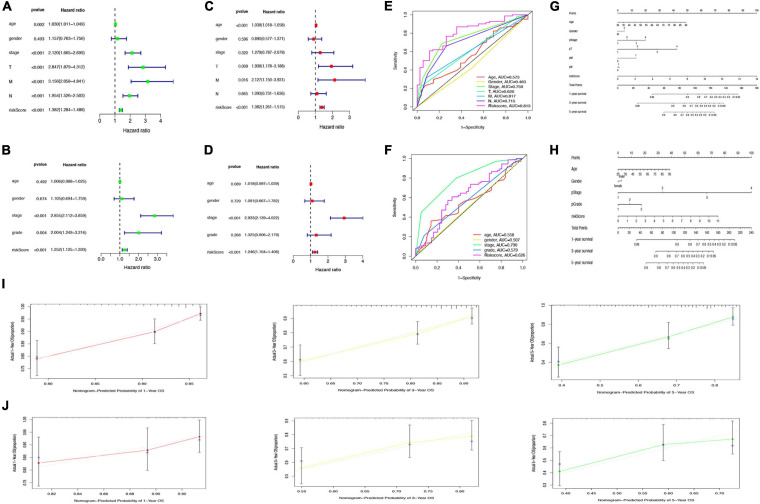
Prediction of overall survival (OS) by risk score and clinical characteristics. **(A,B)** Forest plot of risk scores and other clinical factors based on a univariate Cox regression analysis in The Cancer Genome Atlas (TCGA) **(A)** and GSE17536 cohort **(B)**. **(C,D)** Forest plot of risk scores and other clinical factors based on a multivariate Cox regression analysis in TCGA **(C)** and GSE17536 **(D)** cohort. **(E,F)** Comparison of specificity and sensitivity of 5-year overall survival rate between different clinical traits and GRG risk models in TCGA **(E)** and GSE17536 **(F)** cohort. **(G,H)** Nomogram with a combination of risk score and different clinical traits in TCGA **(G)** and GSE17536 **(H)** cohort. **(I,J)** A calibration plot for predicting the accuracy of the nomogram in TCGA **(I)** and GSE17536 **(J)** cohort.

Univariate Cox regression analysis revealed the significant association of risk score with OS in both TCGA cohort (hazard ratio [HR] = 1.382, 95% confidence interval [CI] = 1.284–1.488, *p* < 0.001) and GSE17536 cohort (HR = 1.252, 95% CI = 1.125–1.393, *p* < 0.001); Figures 8A,B). Multivariate Cox regression analysis with correction for distinct confounding parameters still revealed the risk score as an independent predictor for OS (TCGA cohort: HR = 1.382, 95% CI = 1.261–1.515, *p* < 0.001; GSE 17536 cohort: HR = 1.246, 95% CI = 1.104–1.406, *p* < 0.001; Figures 8C,D). Subsequently, ROC analysis was used to explore whether the risk score model was more accurate in predicting OS than clinical characters. As shown in Figures 8E,F, the predictive ability of the risk score model was stronger than that of other clinical characteristics. To establish a quantitative tool capable of predicting the clinical application of OS in patients with colon cancer, we established a nomogram integrating the risk score and clinical characters to calculate 1-, 3-, and 5-year OS rates of patients (Figures 8G,H). Calibration plots indicated that the nomogram versus an ideal model showed high consistency in TCGA and GSE17536 cohorts (Figures 8I,J).

### Identification of Potent Drugs Targeting Glycolysis-Related Genes in the CMap Database

**FIGURE 9:**
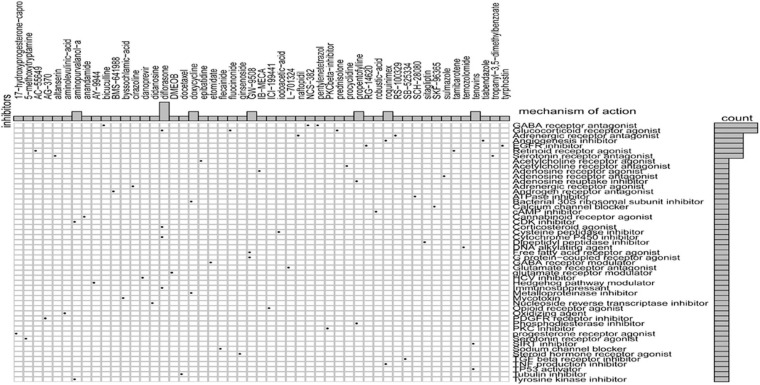
CMap database analysis identifies novel candidate drugs targeting the glycolysis-related gene (GRG)-related signature.

Sixty potential small molecule drugs targeting genes for GRGs were identified by CMap database analysis (Figure 9). The mechanisms of the drugs were distinct. Among the 60 drugs, three (bicuculline, NCS-382, and pentylenetetrazol) are gamma aminobutyric acid (GABA) receptor antagonists and three other drugs (prednisolone, diflorasone, and fluocinonide) are glucocorticoid receptor agonists. The findings indicated that drugs targeting the signature GRGs might have therapeutic importance in the treatment of colon cancer.

### Validation of the Expression and the Prognosis of Eight Glycolysis-Related Genes

**FIGURE 10:**
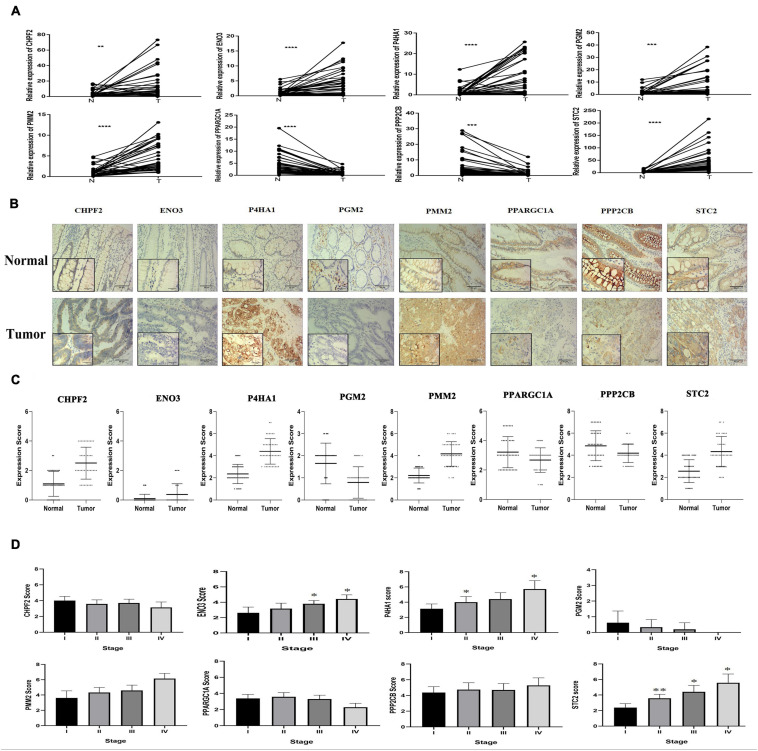
Eight glycolysis-related gene GRG expression in clinical samples. **(A)** mRNA expression of eight GRGs in clinical samples. **(B)** Protein expression analysis of eight GRGs in tumor and normal samples using immunohistochemistry analysis. Scale bar represents × 100, × 400, or 100 **μ**m. **(C)** Eight GRG protein scores in tumor and normal samples. **(D)** GRG protein expression at the distinct stage of tumor. One-way analysis of variance used for statistical analysis, *p < 0.05, **p < 0.01 versus stage I. All data are expressed as mean ± SEM.

qRT-PCR was performed from RNA extracted from 43 pairs of colon cancer and adjacent normal tissues to observe the relative expression levels of eight GRGs (P4HA1, STC2, CHPF2, PMM2, PGM2, ENO3, PPARGC1A, and PPP2CB). P4HA1, STC2, CHPF2, PMM2, PGM2, and ENO3 were expressed at higher levels in COAD tissues, with lower expression of PPP2CB and PPARGC1A (Figure 10A). The findings were consistent with the results of analyses of the Gene Expression Profiling Interactive Analysis (GEPIA) and UALCAN online databases (Supplementary Figure 3).

Similarly, immunohistochemistry (IHC) staining of colon cancer tissue with antibodies to P4HA1, STC2, CHPF2, PMM2, PGM2, ENO3, PPARGC1A, and PPP2CB had higher expression of the P4HA1, STC2, PMM2, CHPF2, and ENO3 GRGs in COAD tissue and lower expression of the PPP2CB, PPARGC1A, and PGM2 GRGs in the same tissues (Figures 10B,C). In addition, there were significant differences in the expression of STC2 in the COAD tissues at different stages of COAD (I, II, III, and IV; Figure 10D). These findings suggest that the expression level of STC2 changes with the progression of tumors.

To further explore the potential prognostic value of eight GRGs in COAD patients, we performed the Kaplan–Meier survival analysis to determine the effect of the eight GRGs on OS in TCGA COAD patients. Overexpression of STC2, ENO3, P4HA1, and CHPF2 and low expression of PPP2CB, PPARGC1A, PMM2, and PGM2 are associated with lower OS rate in COAD patients (Supplementary Figure 4).

### Knockout of STC2 Protein Inhibits the Proliferation and Invasion of Colorectal Cancer Cells

**FIGURE 11:**
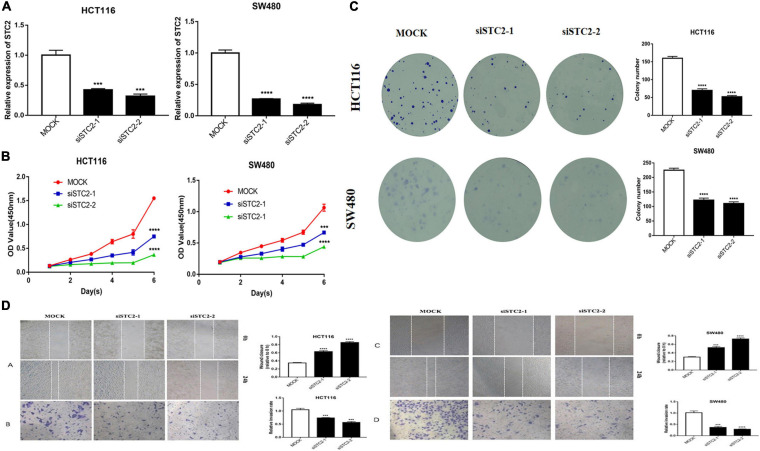
STC2 knockdown inhibits cell proliferation, migration, and invasion *in vitro*. **(A)** STC2 expression in STC2-siRNA HCT-116 and SW-480 cells. **(B)** Growth curves for HCT-116 and SW-480 cells after transfection with STC2 siRNA determined by Cell Counting Kit-8 (CCK-8) analysis. **(C)** Clonogenic assay on STC2-siRNA knockdown HCT-116 and SW-480 cell lines showing decreased colony formation compared with the negative control. **(D)** Decreased cell migration and Transwell invasion activity in STC2-siRNA knockdown HCT-116 and SW-480 cells. Results are expressed as mean ± SEM. Significance difference was tested by one-way ANOVA: ***p < 0.001 versus mock, ****p < 0.0001 versus mock.

Considering the potential role of STC2 in CRC progression, we performed *in vitro* proliferation, colony formation, migration, and invasion assays. The high expression of STC2 was associated with poor outcomes of CRC patients. We further evaluated the effect of STC2 depletion on the cellular process and BP in the CRC cells. Functionally, knockdown of STC2 by siRNA resulted in decreased cellular proliferation and colony formation by HCT116 and SW480 cells (Figures 11A,B). Similarly, knockdown of STC2 in HCT116 and SW480 cells using short hairpin RNA decreased cellular migration and invasion compared with control cells (Figures 11C,D). The findings confirmed the potential role of STC2 in the progression or CRC.

## Discussion

The present analyses revealed 226 DE-GRGs in TCGA COAD patients. In addition, gene ontology and KEGG analyses of DE-GRGs revealed their involvement in regulating the distinct functions and pathways involved in abnormal energy metabolism during tumor development and metastasis. Furthermore, eight GRGs were identified as the most robust glycolysis-related prognostic signature. This signature was used to categorize the colon cancer patients into the low- and high-risk groups with significant differences in survival outcomes. Similarly, univariate and multivariate Cox regression analyses of eight GRGs demonstrated the independent ability to predict patient prognosis. The results further indicate that the signature GRGs are closely related to many clinical characteristics, such as the increased risk score with tumor development and distant metastasis. This discovery could aid in the dynamic monitoring of colon cancer patients. In addition, we further confirmed the expression of eight GRGs in colon cancer by qRT-PCR and IHC analyses. Surprisingly, the expression of protein increased with increased disease stage. Cell cloning and invasion experiments further confirmed the tumor-promoting function of STC2.

To explore the underlying mechanisms of the GRGs model, GSEA was performed to explore KEGG pathways among the two risk groups. Aberrant activation of distinct signaling pathways, including the notch signaling pathway, mitogen-activated protein kinase signaling pathway, regulation of actin cytoskeleton, phosphatidylinositol, and the transforming growth factor-beta signaling pathway, among the high-risk score patients is associated with poor prognosis of COAD (Meng et al., 2009; Lähde et al., 2020).

Considering the heterogeneity and complexity in tumor development, we comprehensively analyzed glycolysis-related models, including the TME, epigenetics, and tumor stemness index, to better understand the potential underlying mechanism among the low- and high-risk groups of COAD patients. Analysis of immune cell infiltration revealed a significant difference between the risk groups, suggesting that abnormal glycolysis metabolism could alter the immune microenvironment, which could affect prognosis and treatment. A prior study (Song and Wu, 2020) reported an association between decreased infiltration of CD4+ memory resting T cells and poor prognosis. Other studies reported a rapid transformation of resting dendritic cells to activated dendritic cells under *in vivo* stimulation and their participation in adaptive immune responses (Joffre et al., 2009). The number of infiltrating dendritic cells has been positively associated with survival in colon cancer and pancreatic ductal adenocarcinoma (Nagorsen et al., 2007; Wu et al., 2020). In addition, the increase of M0 macrophages can lead to decreased immune activity and poor prognosis in digestive system tumors (Nagorsen et al., 2007; Nie et al., 2020). Considering the significant role of GRGs in the development and prognostic of colon cancer, we analyzed the differences between TMB, CNV, and mRNAsi in the low- and high-risk groups of COAD patients. Patients with high-risk score had higher TMB; however, there was no significant difference in total TMB. Higher tumor stem cell index and abnormal CNV were observed in the high-risk group, strongly implicating GRGs as the genetic alteration.

Similarly, scrutiny of the GDSC database determined the effectiveness of different chemotherapeutic drugs in the treatment of low- and high-risk COAD patients. Based on the IC50 values, olaparib, veliparib, axitinib, metformin, and rapamycin displayed a better response in treating the low-risk score COAD patients. These drugs have been reported to be important in the treatment of colon cancer patients (Faller et al., 2015; Arena et al., 2020; Guo et al., 2020). Additionally, using the CMap database, we identified 60 small molecule drugs with therapeutic potential. These drugs may have pronounced efficacy in COAD treatment. The drugs include glucocorticoid receptor agonist (prednisolone, diflorasone, and fluocinonide), GABA receptor antagonists (bicuculline, NCS-382, and pentylenetetrazol), inhibitors of angiogenesis (roquinimex and tiabendazole), and EGFR inhibitors (tyrphostin and RG-14620). Identification of distinct candidate drugs might be useful in the treatment of COAD patients by targeting GRGs. Together, the findings clearly indicate the significant importance of the eight GRG genes in COAD prognosis and treatment.

## Conclusion

This comprehensive multi-database study is the first to investigate the expression profile of GRGs and their clinical significance in patients with colon cancer. The formulated risk model may provide an effective clinical tool in the prognosis and optimization of treatment for patients with colon cancer.

## Data Availability Statement

The original contributions presented in the study are included in the article/Supplementary Material, further inquiries can be directed to the corresponding authors.

## Ethics Statement

The studies involving human participants were reviewed and approved by the Shanghai Tenth people’s Hospital of Tongji University of Medicine Shanghai. Written informed consent for participation was not required for this study in accordance with the national legislation and the institutional requirements. Written informed consent was not obtained from the individual(s) for the publication of any potentially identifiable images or data included in this article.

## Author Contributions

ZC contributed to the research design and data analysis. GS performed the experiments and drafted the manuscript. RmB performed the experiments and assisted in editing. RjB assisted in editing. JL and MZ collected the clinical samples. FS assisted in modifying the manuscript and collating references. ZL and SZ revised the manuscript and provided writing guidance. All authors contributed to manuscript revision and read and approved the submitted version.

## Conflict of Interest

The authors declare that the research was conducted in the absence of any commercial or financial relationships that could be construed as a potential conflict of interest.

## Publisher’s Note

All claims expressed in this article are solely those of the authors and do not necessarily represent those of their affiliated organizations, or those of the publisher, the editors and the reviewers. Any product that may be evaluated in this article, or claim that may be made by its manufacturer, is not guaranteed or endorsed by the publisher.

## Footnotes

1
https://portal.gdc.cancer.gov/repository

2
https://string-db.org/

3
http://apps.cytoscape.org/apps/iregulon

4
https://cibersort.standford.edu/

5
https://www.cancerrxgene.org

6
https://clue.io/query

## Supplementary Material

Supplementary Figure 1 Identification of GRGs. GSEA identification of five gene sets that are significantly enriched, including Biocarta Glycolysis Pathway, Glycolytic Process, Hallmark Glycolysis, KEGG Glycolysis Gluconeogenesis, and Reactome Glycolysis.

Supplementary Figure 2 Relationship of GRGs signature with clinicopathological characteristics in GSE17536. **(A)** Box plots showing risk score distribution of different clinical characteristics of COAD tumors in GSE17536 cohort. **(B)** GRGs risk model is of good value in predicting the OS rate of patients with colon cancer in multiple clinical subgroups.

Supplementary Figure 3 Expression of the eight signature GRGs in online databases. **(A)** UALCAN online database (http://ualcan.path.uab.edu/) expression of eight glycolysis related genes in TCGA. **(B)** GEPIA online database (http://gepia.cancer-pku.cn/) expression of eight glycolysis related genes in TCGA and GTEx.

Supplementary Figure 4 Evaluation of eight glycolysis genes on the prognosis of colon cancer.

Supplementary Table 1 Primer sequences of eight glycolysis related genes.

Supplementary Table 2 GO analysis of genes involved in differential glycolysis.

Supplementary Table 3 Analysis of related KEEG pathways involved in differential glycolysis genes.

Supplementary Table 4 Glycolysis related genes are differentially expressed in colon cancer tissues.
