# Supplementary figures and images for: Comprehensive Analysis of Glycolysis-Related Genes for Prognosis, Immune Features, and Candidate Drug Development in Colon Cancer
Source: Front Cell Dev Biol. 2021 Aug 6;9:684322. doi: 10.3389/fcell.2021.684322 (PMC8377503; doi:10.3389/fcell.2021.684322)

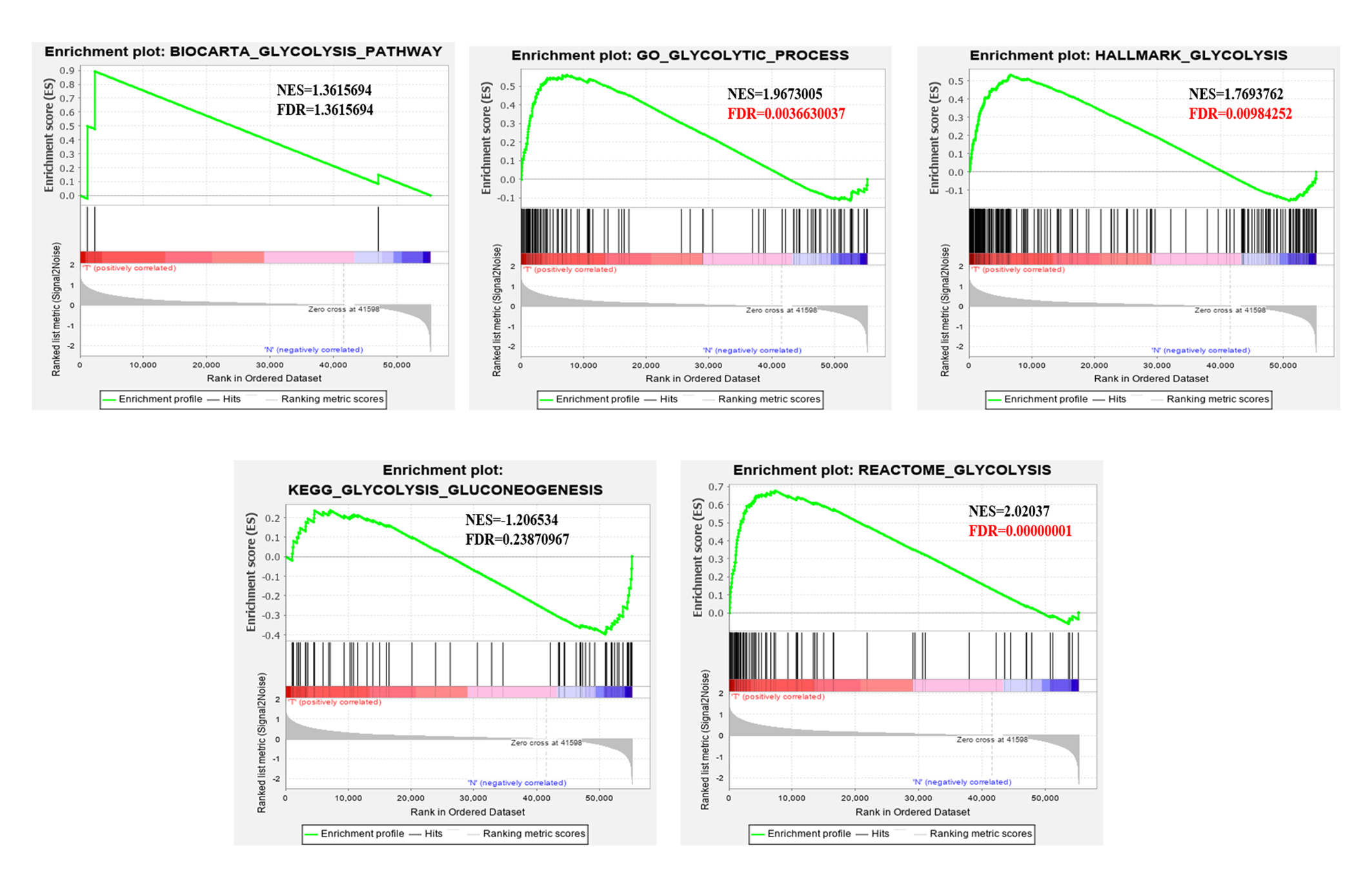

Supplement: Supplementary Figure 1 — Identification of GRGs. GSEA identification of five gene sets that are significantly enriched, including Biocarta Glycolysis Pathway, Glycolytic Process, Hallmark Glycolysis, KEGG Glycolysis Gluconeogenesis, and Reactome Glycolysis. [file Image_1.JPEG]

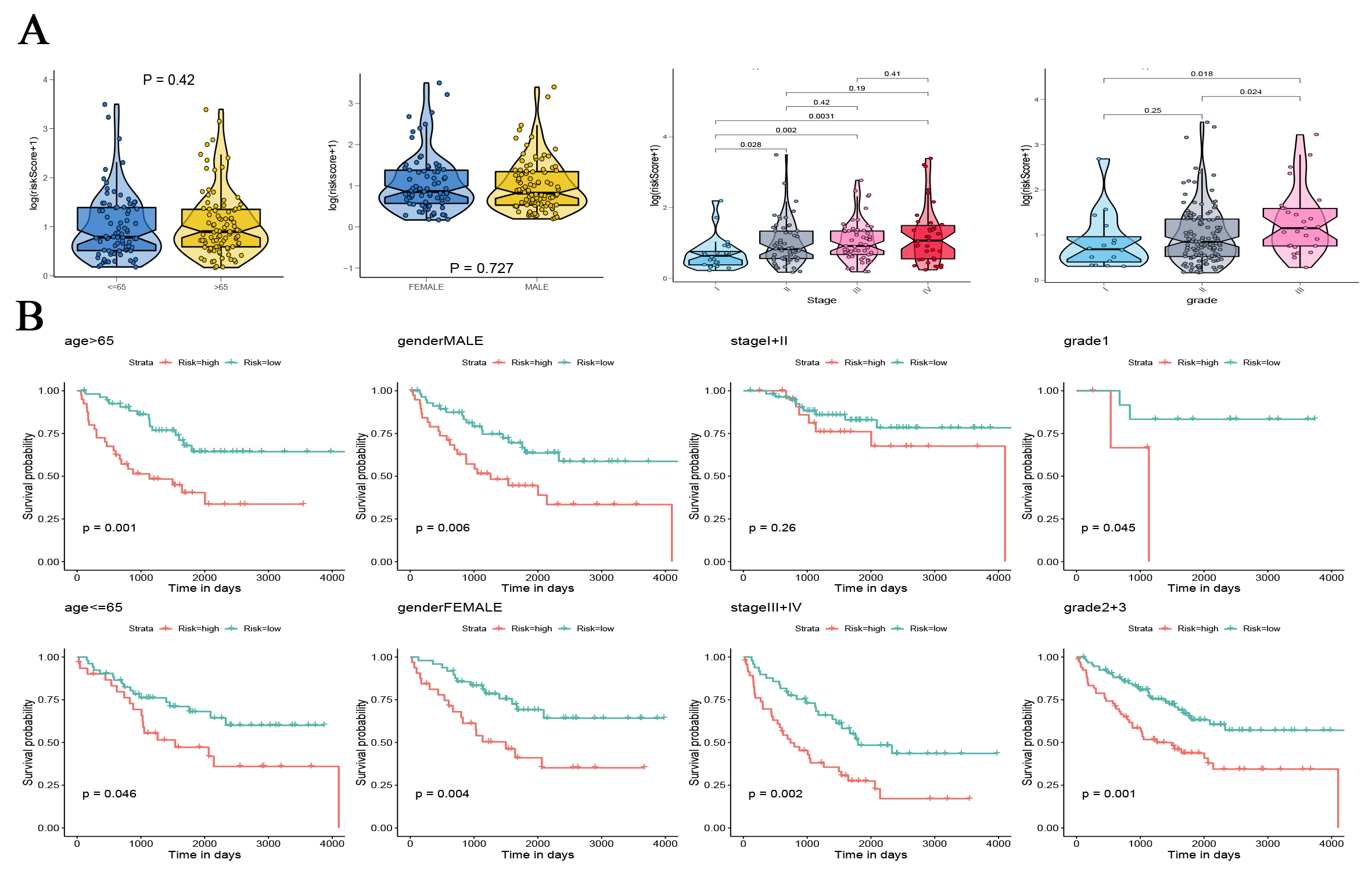

Supplement: Supplementary Figure 2 — Relationship of GRGs signature with clinicopathological characteristics in GSE17536. (A) Box plots showing risk score distribution of different clinical characteristics of COAD tumors in GSE17536 cohort. (B) GRGs risk model is of good value in predicting the OS rate of patients with colon cancer in multiple clinical subgroups. [file Image_2.JPEG]

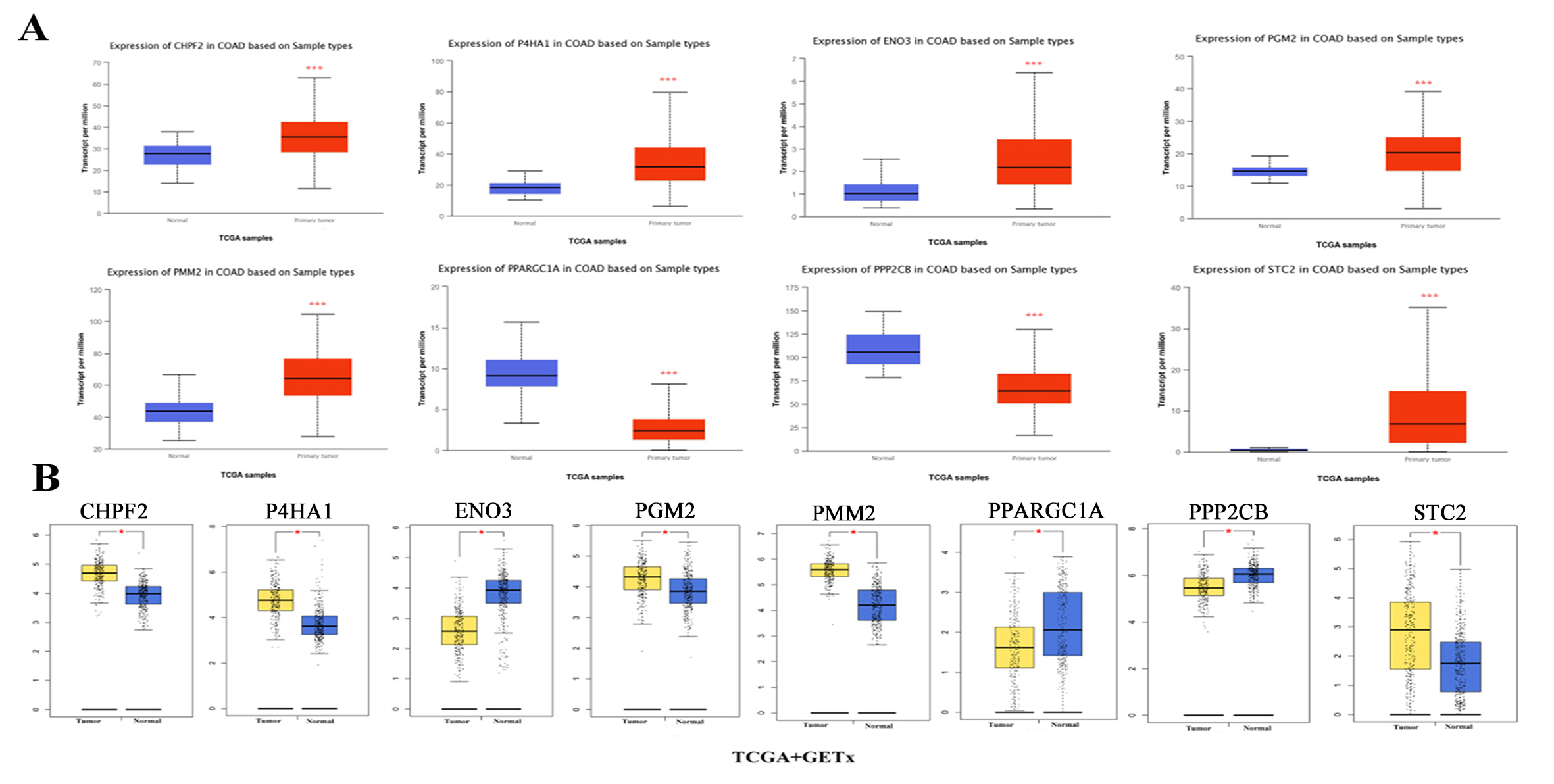

Supplement: Supplementary Figure 3 — Expression of the eight signature GRGs in online databases. (A) UALCAN online database (http://ualcan.path.uab.edu/) expression of eight glycolysis related genes in TCGA. (B) GEPIA online database (http://gepia.cancer-pku.cn/) expression of eight glycolysis related genes in TCGA and GTEx. [file Image_3.JPEG]

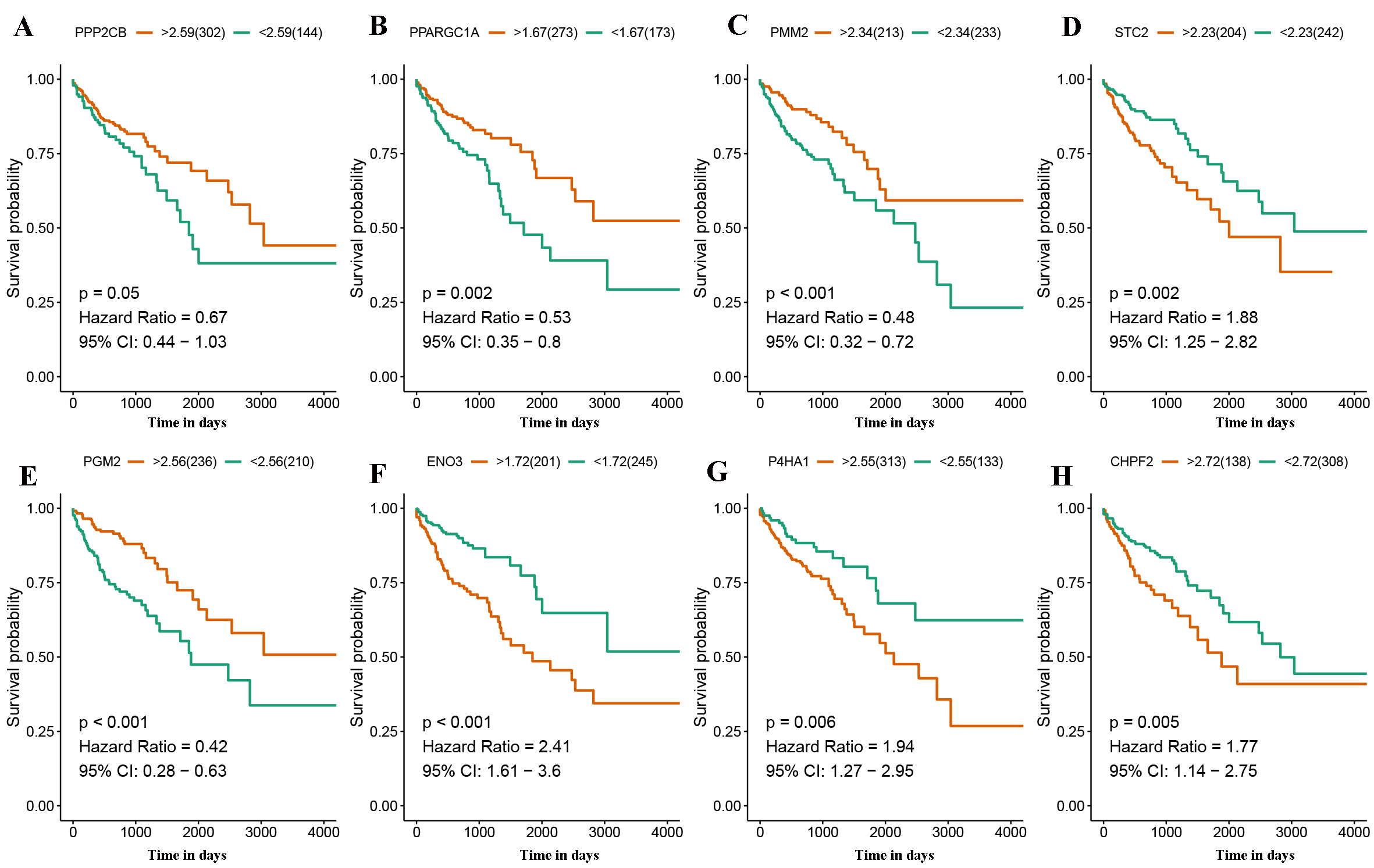

Supplement: Supplementary Figure 4 — Evaluation of eight glycolysis genes on the prognosis of colon cancer. [file Image_4.JPEG]
